# Comparison of the Persistence of Anti-TNF Agents and Ustekinumab in Patients with Crohn’s Disease: A Study Based on the Korean National Database

**DOI:** 10.3390/jcm12062397

**Published:** 2023-03-20

**Authors:** Gi Hyeon Seo, Sung Hoon Jung

**Affiliations:** 1 Department of Healthcare Review and Assessment Committee, Health Insurance Review and Assessment Service, Ministry of Health and Welfare, Wonju 26465, Republic of Korea; 2 Department of Internal Medicine, Eunpyeong St. Mary’s Hospital, The Catholic University of Korea, Seoul 03312, Republic of Korea

**Keywords:** persistence, Crohn’s disease, ustekinumab

## Abstract

Background: Biologics play an important role in the treatment of moderate to severe Crohn’s disease (CD). Ustekinumab was approved for such patients in the Republic of Korea on 1 December 2018. Therefore, we need to compare the efficacy of ustekinumab and anti-TNF inhibitors. Methods: We compared one-year persistence rates between anti-TNF inhibitors and ustekinumab in moderate-to-severe CD patients using Korean National Health Insurance Service data from 1 December 2016 to 30 November 2021. We also analysed the risk factors for the non-persistence of biologics. Results: The one-year persistence rates with index therapy in bio-naïve and bio-experienced patients were 87.7% and 69.7% for infliximab (*p* < 0.001), 85.1% and 72.8% for adalimumab (*p* < 0.001), and 92.1% and 89.8% for ustekinumab (*p* = 0.333), respectively. The risk factors for non-persistence were older age, non-use of an immune modulator, and previous biologic exposure in both the infliximab and adalimumab groups. The one-year persistence rate of ustekinumab was higher than that of anti-TNF inhibitors in bio-naïve patients (hazard ratio [HR] 0.53; 95% confidence interval [CI] 0.35–0.81; *p* = 0.003) and bio-experienced patients (HR 0.32; 95% CI 0.22–0.45; *p* < 0.001). Conclusions: Ustekinumab was superior in bio-naïve CD patients compared to anti-TNF inhibitors. However, the follow-up time was relatively short; further studies should continuously collect and analyse data.

## 1. Introduction

Crohn’s disease (CD) is a chronic relapsing and remitting inflammatory condition that affects the gastrointestinal tract. The number of patients with CD has recently increased in the Republic of Korea and other Asian countries [1,2,3]. Treatment of moderate-to-severe CD is based on immunosuppression with steroids, immune modulators, and biologics. Among these agents, biologics play a particularly important role in the treatment of CD [4]. Anti-tumour necrosis factor (TNF) inhibitors (infliximab and adalimumab) have proven effective for inducing and maintaining remission in patients with moderate-to-severe CD [5,6] and are available for this population in the Republic of Korea.

Biologics are being gradually introduced in the Republic of Korea. Vedolizumab was authorized for use as a second-line treatment for CD patients on 1 August 2017, following the approval of anti-TNF medicines (infliximab and adalimumab) and then as a first-line treatment on 1 August 2020. Ustekinumab was approved for such patients in the Republic of Korea on 1 December 2018. 

Ustekinumab is a monoclonal antibody for the p40 subunit of interleukin (IL)-12 and IL-23 approved for the treatment of CD by the US Food and Drug Administration and the European Medicines Agency [7]. The efficacy and safety of ustekinumab in adults with moderate-to-severe CD have been evaluated in clinical studies [8,9,10,11].

Although some studies have evaluated and compared the efficacy of biologics, it is difficult to directly apply these results to clinical practice. Therefore, real-world data for biologics are used to guide decision-making. Non-persistence of biologics occurs due to a loss of response, adverse events, or poor adherence [12,13]. A recent Korean study reported similar persistence rates among the anti-TNF inhibitors used for CD [14]. However, comparisons with ustekinumab in a real-world setting are lacking. In addition, genetic differences between Eastern and Western populations, as well as differences in health insurance systems, can affect drug persistence.

Therefore, this study was conducted to compare the persistence between anti-TNF inhibitors and ustekinumab in moderate-to-severe CD patients. We also analyzed the risk factors for non-persistence of biologics in a real-world setting.

## 2. Materials and Methods

### 2.1. Data Source

We extracted International Classification of Diseases, 10th revision (ICD-10) diagnostic codes from the National Health Insurance Service (NHIS) database, as well as V codes from the Rare Intractable Diseases (RID) database. For RID registration of CD patients in the Republic of Korea, clinical, endoscopic, and pathological diagnoses of CD are required. This study was approved by the Institutional Review Board of the Health Insurance Review & Assessment Service (HIRA 2022-054).

### 2.2. Patient Selection

We enrolled CD patients (identified by ICD-10 code K50.x and V code V130 in the NHIS database) from 1 December 2018 to 30 November 2020. The search was restricted to CD patients for whom treatment was initiated with a new anti-TNF inhibitor (infliximab or adalimumab) or ustekinumab. All enrolled patients were analysed from 1 December 2016, to 30 November 2021. The HIRA permits the use of biologics for patients with moderate-to-severe inflammatory bowel disease (IBD) who are not responsive, intolerant, or contraindicated to conventional drugs, such as aminosalicylates (ASA), corticosteroids, and immune modulators. All Korean physicians adhere to this policy; therefore, all enrolled patients had moderate-to-severe CD.

### 2.3. Outcomes

The patient demographics were summarised on the date on which the anti-TNF inhibitor or ustekinumab was initiated. We collected data on IBD medications, including 5-ASA, immunomodulators (azathioprine, mercaptopurine, cyclosporine, tacrolimus, and methotrexate), steroids, and biologics (infliximab, adalimumab, and ustekinumab). We defined the use of conventional medications, such as immunomodulators and steroids, as having occurred if the individual had used the medication for at least four weeks during the year prior to the initiation of the biological agent.

We defined persistence as not discontinuing or switching the drug. Drug switching during the follow-up period was assumed to have occurred if another biologic was prescribed. Drug discontinuation was assumed to have occurred if the index agent was not prescribed for >90 days. Bio-naïve patients were those without for any biologic in the 24 months before the index date, and bio-experienced patients were those who received one or more than one line of biologics, respectively.

The one-year persistence rates were compared between patients administered an anti-TNF inhibitor and those administered ustekinumab. The risk factors for non-persistence for each biologic (infliximab, adalimumab, and ustekinumab) were investigated.

### 2.4. Statistics

The *t*-test and chi-square test were used as appropriate to compare data between the two groups. Continuous variables are presented as means ± standard deviations, and categorical variables are presented as numbers with percentages. We used Kaplan-Meier curves to compare the one-year persistence rates. All statistical tests were two-tailed, and a *p*-value <0.05 was considered significant. All analyses were performed using R software (ver. 3.4.3; R Foundation for Statistical Computing, Vienna, Austria).

## 3. Results

### 3.1. Demographic Characteristics

We enrolled 2987 CD patients who were starting biologics (infliximab, *n* = 1343; adalimumab, *n* = 756; ustekinumab, *n* = 888) from 1 December 2018 to 30 November 2020 and reviewed their medical records between 1 December 2016 and 30 November 2021. The mean age of the ustekinumab group was higher than of the infliximab and adalimumab (anti-TNF inhibitor) groups (*p* < 0.001). The disease duration was longer in the ustekinumab than anti-TNF inhibitor groups (*p* < 0.001). There were 2152 bio-naïve patients (infliximab, *n* = 1267 [94.3%]; adalimumab, *n* = 583 [77.1%]; ustekinumab, *n* = 302 [34.0%]). The number of bio-experienced patients was higher in the ustekinumab than anti-TNF inhibitor groups (*p* < 0.001). The other patient characteristics are summarised in Table 1.

### 3.2. Comparison of the One-Year Persistence Rates

**Figure 1:**
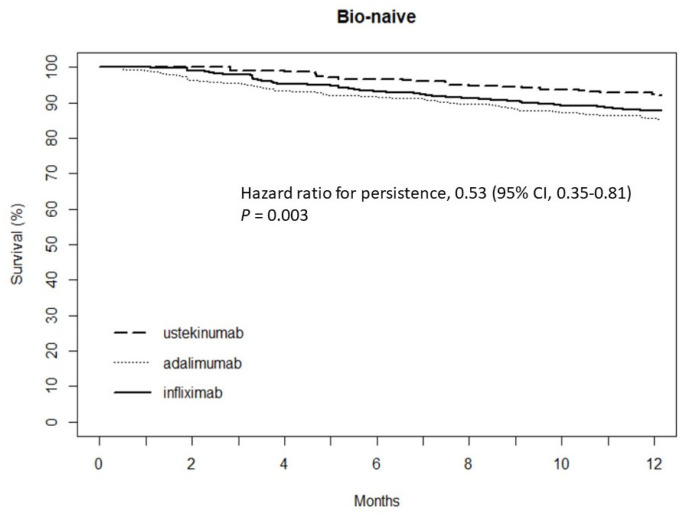
Comparison of persistence between anti-TNF inhibitors and ustekinumab in bio-naïve CD patients.

**Figure 2:**
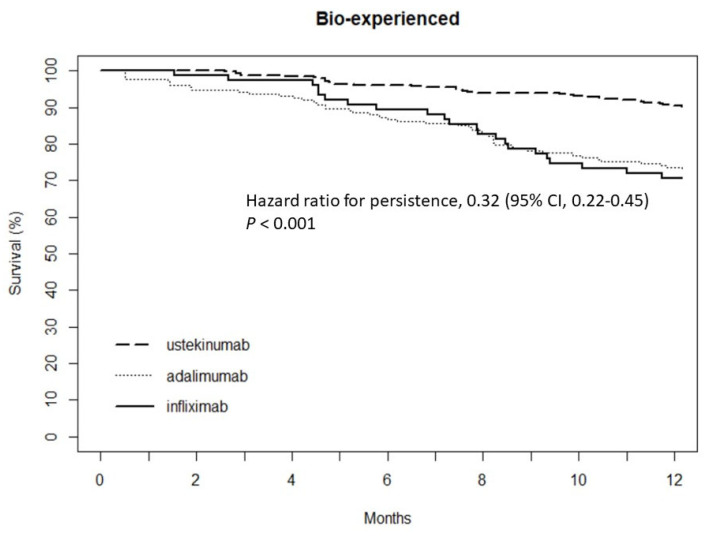
Comparison of persistence between anti-TNF inhibitors and ustekinumab in bio-experienced CD patients.

Overall, the one-year persistence rate for the biologics was 2590 (86.7%) (bio-naïve, *n* = 1885; bio-experienced, *n* = 705). The one-year persistence rates with index therapy in bio-naïve and bio-experienced patients were 87.7% and 69.7% for infliximab (*p* < 0.001), 85.1% and 72.8% for adalimumab (*p* < 0.001), and 92.1% and 89.8% for ustekinumab (*p* = 0.333), respectively. A significant difference was detected between the anti-TNF inhibitors (infliximab and adalimumab) and ustekinumab in the one-year persistence rate for bio-naive patients (hazard ratio [HR] 0.53; 95% confidence interval [CI] 0.35–0.81; *p* = 0.003; Figure 1). The one-year persistence rate of ustekinumab was higher than that of the anti-TNF inhibitors in bio-experienced patients (HR 0.32; 95% CI 0.22–0.45; *p* < 0.001; Figure 2).

### 3.3. Risk Factors for Non-Persistence

Discontinuation and switching of the index therapy were considered to indicate non-persistence, and they occurred in 131 (9.8%) and 48 (3.6%) patients taking infliximab, 118 (15.6%) and 16 (2.1%) patients taking adalimumab, and 45 (5.1%) and 39 (4.4%) patients taking ustekinumab, respectively. The risk factors for non-persistence were older age, non-use of an immune modulator, and previous biologic exposure in both the infliximab and adalimumab groups (Table 2 and Table 3). No significant risk factors for non-persistence were observed in the ustekinumab group (Table 4).

## 4. Discussion

The number of CD patients is increasing in Asia, including the Republic of Korea, and biologics are being gradually introduced. Thus, real-world data on which drugs are most effective over extended periods are needed. Four biologics (infliximab, adalimumab, ustekinumab, and vedolizumab) have been approved for CD in the Republic of Korea. In this study, we compared the persistence of these biologics (except vedolizumab) to that of anti-TNF inhibitors. Vedolizumab was excluded from the comparison of persistence because it was initially approved only for bio-experienced IBD patients.

Overall, the one-year persistence rate of the biologics in our study was 86.7%, which was higher than that of studies conducted in other countries [15,16]. In this study, the one-year persistence rates of the anti-TNF inhibitors in CD patients were 86.7% for infliximab and 82.3% for adalimumab. A recent German claims data analysis showed that the one-year persistence rate of anti-TNF inhibitors was 60.1% in patients with IBD [15]. In the Persistence Australian National IBD Cohort study, the one-year persistence rates of anti-TNF inhibitors were 68.1% for infliximab and 64.2% for adalimumab in patients with luminal CD, as well as 79.7% for infliximab and 75.7% for adalimumab in patients with fistulizing CD. A recent Danish nationwide cohort study found that the one-year persistence rates of anti-TNF inhibitors were 61.5% for infliximab and 58.1% for adalimumab in CD patients. Similarly, the one-year persistence rates of anti-TNF inhibitors used as second-line treatments were 50.4% for infliximab and 49.3% for adalimumab in CD patients [17]. However, the one-year persistence rates in the Republic of Korea for anti-TNF inhibitors are 86.2% for infliximab and 84.9% for adalimumab; these latter data closely corresponded with our results [14]. One of the reasons for the differences between these studies may be differences in health insurance systems among countries. In the Republic of Korea, the HIRA allows the use of biologics in moderate-to-severe CD cases, and it recommends that physicians continue to prescribe the index drug for at least six months if the patient initially responds. Secondly, fewer biologics are available in the Republic of Korea (*n* = 4) than in the USA and Europe. Thirdly, genetic differences could affect the response to biologics. The sustained response and remission rates of infliximab in a real-life cohort of Korean patients with CD were high [18]. More studies are needed on this topic.

In this study, the overall one-year persistence rate was 90.5% for ustekinumab in CD patients. This rate was higher than that reported by Western studies [16,19]. The difference can be explained by the differences in anti-TNF inhibitors. In particular, anti-TNF inhibitor-experienced CD patients in Republic of Korea have few choices with respect to changing biologics. Therefore, in this study, although ustekinumab was slightly superior in bio-naïve CD patients compared to the anti-TNF inhibitors in terms of the one-year persistence rate, the one-year persistence rate of ustekinumab for bio-experienced CD patients was higher than that of patients taking an anti-TNF inhibitor. In addition, because ustekinumab is an anti-IL-12 and anti-IL23 monoclonal antibody, it has a different mechanism of action and could be more effective for bio-experienced CD patients.

The risk factors for the non-persistence of anti-TNF inhibitors in the CD patients in this study were old age, non-use of an immunomodulator, and previous exposure to biologics. This is similar to previous studies reporting that first-line therapy and immunomodulator co-therapy were associated with persistence of biological agents [16]. However, no risk factors for non-persistence of ustekinumab were observed in our CD patients. A recent study of the persistence rate of ustekinumab showed that it did not differ between CD patients receiving monotherapy and those receiving ustekinumab combined with an immunomodulatory [19].

Interestingly, our study found that patients in the ustekinumab group had higher mean age and disease duration. Several explanations are possible for this finding. Firstly, younger individuals with Crohn’s disease may experience more severe symptoms than older individuals because the disease can be more aggressive in its early stages. Thus, some younger patients may be prescribed anti-TNF inhibitors, which are traditionally used as a first-line treatment for Crohn’s disease. However, ustekinumab has also been shown to be effective in treating the condition, since it targets a different immune system pathway than anti-TNF inhibitors. Secondly, anti-TNF inhibitors have been in use for a longer time and have a more established safety profile, but they can be associated with risks, such as increased risk of infections and infusion reactions. In contrast, ustekinumab may be associated with a lower risk of infections, which is especially important given the prevalence of tuberculosis in the Republic of Korea. These safety concerns may have influenced physicians’ choice of biologics regardless of Crohn’s disease severity. Thirdly, ustekinumab is a newer medication that has recently been approved for use in Crohn’s disease. Thus, patients receiving ustekinumab in the Republic of Korea may have had a longer prevalence period of Crohn’s disease than those receiving anti-TNF inhibitors, as supported by our finding that more patients with biological experience were in the ustekinumab group.

Moreover, in the bio-experienced group of patients, vedolizumab was present in 14% of the ustekinumab group compared to 0.3–2.2% in the anti-TNF group. Biologics are gradually being introduced in the Republic of Korea, with vedolizumab authorized for use as a second-line treatment for CD patients on 1 August 2017, following the approval of anti-TNF medicines (infliximab and adalimumab), and then as a first-line treatment on 1 August 2020. However, HIRA does not allow patients who have failed biologics to reuse the same drug. Therefore, most of the vedolizumab-experienced patients in this study received anti-TNF inhibitors and were enrolled in the ustekinumab group as bio-experienced patients.

Nevertheless, it is important to note that the choice of biologics for treating Crohn’s disease is based on multiple factors, such as disease severity, inflammation location, medical history, and the potential risks and benefits of each medication, rather than solely on the prevalence period of the disease. The selection of medication for treating Crohn’s disease is individualized and varies between patients and different healthcare systems.

This study also revealed an intriguing finding: the proportion of women (29%) was unexpectedly low, while the proportion of patients treated with 5-ASA (77%) was surprisingly high. This may be partly explained by the fact that men are twice as likely to develop Crohn’s disease in the Republic of Korea, as demonstrated by several studies conducted by the Korean NHIS [20,21]. Secondly, it is possible that men are more likely to develop aggressive forms of Crohn’s disease at a younger age than women [22], leading to a higher proportion of men being treated with biologics. Regarding the use of 5-ASA, it should be noted that the HIRA allows the use of biologics for patients with moderate-to-severe IBD who are not responsive to, intolerant to, or contraindicated to conventional drugs, such as 5-ASA, corticosteroids, and immune modulators. All Korean physicians follow this policy, which is why the majority of patients have used 5-ASA.

Although this is the first Korean study to compare the persistence of anti-TNF inhibitors and ustekinumab, some limitations should be discussed. Firstly, we could not determine the reasons for discontinuing or switching biologics. As a result, adverse events or adherence may have affected the evaluation of effectiveness. Secondly, no data were available about smoking, disease location, disease activity and severity, or CD phenotypes, including fistulizing disease. Thirdly, we could not investigate the doses of the biologics, including dose escalation during follow-up. Fourthly, there is no available data on co-medications by steroids during follow-up. These points have the potential to affect drug persistency, so attention needs to be paid to the interpretation of the results of this study. Finally, we did not include vedolizumab (VDZ) as an additional comparison group due to changes in drug permits in the Republic of Korea. This may have limited the interpretation of our study’s results on biologic persistency. Unfortunately, further analysis is not possible, as the data analysis period approved by HIRA for this study has ended. Finally, several guidelines may have affected the physicians’ drug choices, which may in turn have affected our results.

## 5. Conclusions

Ustekinumab was superior to anti-TNF inhibitors in bio-native CD patients. Immunomodulatory combined therapy should be recommended when an anti-TNF inhibitor is required. In particular, ustekinumab should be used for anti-TNF inhibitor-experienced CD patients. However, the follow-up time was relatively short; further studies should continuously collect and analyse data.

## Figures and Tables

**Table 1:** Baseline characteristics of the study population, *n* (%).

Variables	Total (*n* = 2987)	Infliximab (*n* = 1343)	Adalimumab (*n* = 756)	Ustekinumab (*n* = 888)
Age at diagnosis	29.9 ± 13.1	26.4 ± 12.8	30.2 ± 12.9	34.9 ± 12.2
Age group				
<30	1715 (57.4)	929 (69.2)	429 (56.7)	357 (40.2)
≥30	1272 (42.6)	414 (30.8)	327 (43.3)	531 (59.8)
Sex				
Male	2111 (70.7)	990 (73.7)	519 (68.7)	602 (67.8)
Female	876 (29.3)	353 (26.3)	237 (31.3)	286 (32.2)
Disease duration				
<1.0 year	990 (33.1)	693 (51.6)	227 (30.0)	70 (7.9)
1.0–2.9	418 (14.0)	179 (13.3)	135 (17.9)	104 (11.7)
3.0–6.9	577 (19.3)	221 (16.5)	155 (20.5)	201 (22.6)
>7.0 year	1002 (33.5)	250 (18.6)	239 (31.6)	513 (57.8)
Follow up duration (month)	11.4 ± 2.3	11.4 ± 2.3	11.0 ± 2.9	11.7 ± 1.7
Medication				
5-ASA	2296 (76.9)	1084 (80.7)	594 (78.6)	618 (69.6)
Immunomodulators	2407 (80.6)	1130 (84.1)	644 (85.2)	633 (71.3)
Steroid	1804 (60.4)	841 (62.6)	486 (64.3)	477 (53.7)
Biologics				
Naïve	2152 (72.0)	1267 (94.3)	583 (77.1)	302 (34.0)
Experienced				
Infliximab	451 (15.1)		156 (20.6)	295 (33.2)
Adalimumab	239 (8.0)	72 (5.4)		167 (18.8)
Vedolizumab	145 (4.9)	4 (0.3)	17 (2.2)	124 (14.0)
One-year outcome				
Persistence	2590 (86.7)	1164 (86.7)	622 (82.3)	804 (90.5)
Discontinuation	294 (9.8)	131 (9.8)	118 (15.6)	45 (5.1)
Switching	103 (3.4)	48 (3.6)	16 (2.1)	39 (4.4)

**Table 2:** Risk factors of non-persistence in infliximab group.

Variables	Persistence 1164 (86.7)	Non-Persistence 179 (13.3)	*p*-Value
Age at diagnosis	25.9 + 12.3	29.8 + 15.1	0.001
Sex			0.363
Male	863 (87.2)	127 (12.8)	
Female	301 (85.3)	52 (14.7)	
Disease duration			0.231
<1.0 year	611 (88.2)	82 (11.8)	
1.0–2.9	151 (84.4)	28 (15.6)	
3.0–6.9	193 (87.3)	28 (12.7)	
>7.0 year	209 (83.6)	41 (16.4)	
Medication			
5-ASA	932 (86.0)	152 (14.0)	0.154
Immunomodulators	994 (88.0)	136 (12.0)	0.002
Steroid	722 (85.9)	119 (14.1)	0.281
Biologics			<0.001
Naïve	1111 (87.7)	156 (12.3)	
Experienced	53 (69.7)	23 (30.3)	

**Table 3:** Risk factors of non-persistence in adalimumab group.

Variables	Persistence 622 (82.3)	Non-Persistence 134 (13.3)	*p*-Value
Age at diagnosis	29.7 + 12.4	32.6 + 14.7	0.036
Sex			0.999
Male	427 (82.3)	92 (17.7)	
Female	195 (82.3)	42 (17.7)	
Disease duration			0.327
<1.0 year	185 (81.5)	42 (18.5)	
1.0–2.9	118 (87.4)	17 (12.6)	
3.0–6.9	128 (82.6)	27 (17.4)	
>7.0 year	191 (79.9)	48 (20.1)	
Medication			
5-ASA	487 (85.1)	107 (18.0)	0.729
Immunomodulators	541 (84.0)	103 (16.0)	0.005
Steroid	402 (82.7)	84 (17.3)	0.691
Biologics			<0.001
Naïve	496 (85.1)	156 (12.3)	
Experienced	126 (72.8)	47 (27.2)	

**Table 4:** Risk factors of non-persistence in ustekinumab group.

Variables	Persistence 804 (90.5)	Non-Persistence 84 (9.5)	*p*-Value
Age at diagnosis	35.1 + 12.2	33.3 + 12.6	0.214
Sex			0.531
Male	542 (90.0)	60 (10.0)	
Female	262 (91.6)	24 (8.4)	
Disease duration			0.327
<1.0 year	61 (87.1)	9 (12.9)	
1.0–2.9	96 (92.3)	8 (7.7)	
3.0–6.9	181 (90.0)	20 (10.0)	
>7.0 year	466 (90.8)	47 (9.2)	
Medication			
5-ASA	563 (91.1)	55 (8.9)	0.385
Immunomodulators	578 (91.3)	55 (8.7)	0.254
Steroid	437 (91.6)	40 (8.4)	0.252
Biologics			0.333
Naïve	278 (92.1)	24 (7.9)	
Experienced	526 (89.8)	60 (10.2)	

## Data Availability

The datasets generated or analyzed during the current study are available from the corresponding author on reasonable request.
